# Universal Principles During Unicuspid Aortic Valve Repair

**DOI:** 10.1016/j.atssr.2026.04.016

**Published:** 2026-04-25

**Authors:** Marek J. Jasinski

**Affiliations:** Department of Cardiac Surgery, Wroclaw Medical University, 213 Borowska, Wroclaw 50 556, Poland

To the Editor

Unicuspid aortic valve (UAV) has been defined as a variant of bicuspid aortic valve (BAV) but with only 1 well-developed commissure. The other 2 commissures are located significantly lower because of their underdevelopment. According to the Sievers classification, it is a variant of BAV type 2. The most recognized concept of UAV repair is bicuspidalization. The neocommissure has to be established, optimally in front of a well-developed noncoronary–left coronary commissure, followed by the reconstruction of leaflets and another commissure. However, it is the tissue replacement that is considered the Achilles' heel of repair, and degeneration of tissue is the most common mechanism of repair failure.

An interesting modification of this approach is presented by Kawabata and colleagues.^1^ The concept is based on preserving the free edge by implanting patches into the leaflet body, enhancing the subcommissural triangle simultaneously. In previous approaches, the dysplastic leaflet tissue is typically reconstructed with a pericardial patch, forming a full body of leaflets around the reconstructed commissure.^2^^,^^3^ In contrast, the Kawabata approach, by preserving the free edge, is probably enhancing tissue durability, confirmed by 6 years of follow-up. Apart from the modified commissural enhancement, the symmetrical commissural orientation achieved by the authors may impose better postoperative durability.^4^ In this regard, the repair is completed with its stabilization by reimplantation into a Dacron prosthesis in a symmetrical 180° configuration.

Interestingly, the same goals can be achieved by using an internal remodeling annuloplasty ring for UAV repair.^5^ The commissural reconstruction is achieved by commissural posts of the ring implanted into the upper half of subcommissural triangles, hence implying a 180° symmetrical configuration, increasing the leaflet’s mobility (Figure). The level of coaptation and neocommissure is also raised by paracommissural plication.

**Figure:**
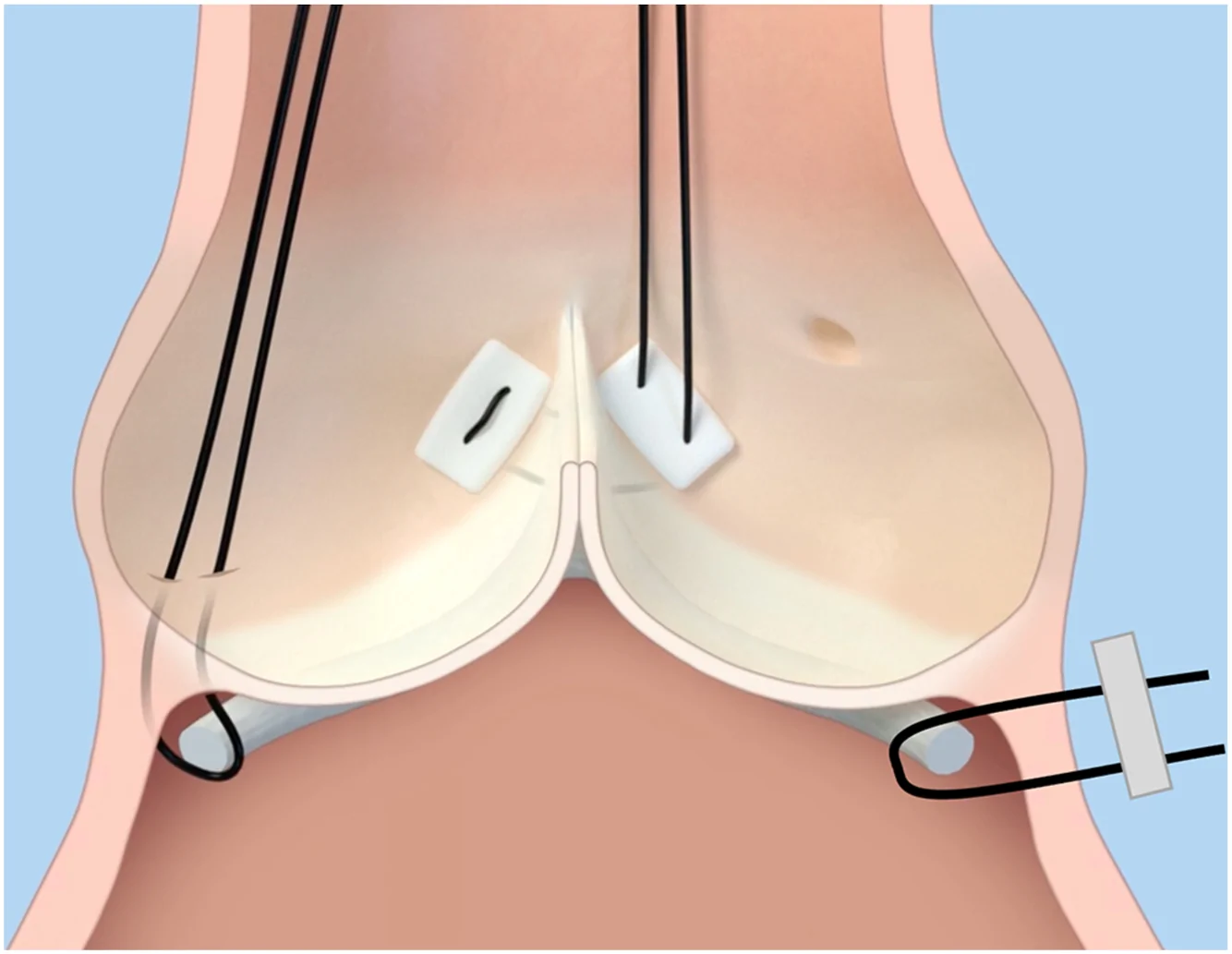
Unicuspid aortic valve repair by symmetrical bicuspidalization and internal annuloplasty neocommissural reconstruction.

These techniques are alternative concepts of repair following the same principles: converting UAV to single-raphe BAV type 1 and repairing the remaining raphe with prolapsing leaflet plication and 180° stabilization with internal remodeling, external annuloplasty, or reimplantation (Figure).
